# Identification and external validation of a prognostic signature associated with DNA repair genes in gastric cancer

**DOI:** 10.1038/s41598-021-86504-8

**Published:** 2021-03-30

**Authors:** Shimin Chen, Wenbo Liu, Yu Huang

**Affiliations:** Department of Gastroenterology, Traditional Chinese Medical Hospital of Taihe Country, No 59, Tuanjie West Road, Taihe County, Fuyang, 236600 Anhui Province China

**Keywords:** Cancer, Computational biology and bioinformatics, Genetics, Biomarkers, Gastroenterology, Oncology

## Abstract

The aim of this study was to construct and validate a DNA repair-related gene signature for evaluating the overall survival (OS) of patients with gastric cancer (GC). Differentially expressed DNA repair genes between GC and normal gastric tissue samples obtained from the TCGA database were identified. Univariate Cox analysis was used to screen survival-related genes and multivariate Cox analysis was applied to construct a DNA repair-related gene signature. An integrated bioinformatics approach was performed to evaluate its diagnostic and prognostic value. The prognostic model and the expression levels of signature genes were validated using an independent external validation cohort. Two genes (CHAF1A, RMI1) were identified to establish the prognostic signature and patients ware stratified into high- and low-risk groups. Patients in high-risk group presented significant shorter survival time than patients in the low-risk group in both cohorts, which were verified by the ROC curves. Multivariate analysis showed that the prognostic signature was an independent predictor for patients with GC after adjustment for other known clinical parameters. A nomogram incorporating the signature and known clinical factors yielded better performance and net benefits in calibration plot and decision curve analyses. Further, the logistic regression classifier based on the two genes presented an excellent diagnostic power in differentiating early HCC and normal tissues with AUCs higher than 0.9. Moreover, Gene Set Enrichment Analysis revealed that diverse cancer-related pathways significantly clustered in the high-risk and low-risk groups. Immune cell infiltration analysis revealed that CHAF1A and RMI1 were correlated with several types of immune cell subtypes. A prognostic signature using CHAF1A and RMI1 was developed that effectively predicted different OS rates among patients with GC. This risk model provides new clinical evidence for the diagnostic accuracy and survival prediction of GC.

## Introduction

Gastric carcinoma (GC) remains the fifth most frequently gastrointestinal malignancies and second leading cause of cancer-related death worldwide, with a high incidence in East Asian countries^1,2^. Despite the rapid therapeutic advances in diagnostic and therapeutic methods, the overall 5-year survival rate remains disappointing. This is due to the fact that patients with early-stage are often asymptomatic, and numerous patients are usually diagnosed at an advanced stage and even with metastatic diseases or relapse, which even combined chemotherapy or radiotherapy fail to bring a favorable outcome^3,4^. Although tumor-node-metastasis (TNM) grading system along with histological subtype is the most commonly used in clinical to predict prognosis and guide treatment decision for GC, it provides not adequate enough prognostic information and cancers with the same TNM stage illustrate differences in clinical outcomes and treatment response^5–7^. Therefore, there is an urgent need to explore novel prognostic biomarkers to increase the accuracy of prognosis prediction.

With the rapid advancement of genome-sequencing technologies, growing evidence has illustrated that gene signatures play key roles in predicting GC prognosis. For example, a classifier combination of five immune genes (CD3, CD274, CD4, PAX5, and GZMB) with age and TNM stage demonstrated better prognostic value than TNM alone, and GC patients with high-risk score presented a favorable prognosis to adjuvant chemotherapy^8^. A recent study constructed an immune-related gene pair signature based on 25 unique genes to predict the prognosis of GC. It was illustrated that patients in high-risk group presented poor prognosis and confirmed in other two independent cohorts, and the signature could use as a predictive tool to identify patients who might benefit from immunotherapy^9^. Recently, gene biomarkers for the diagnosis or prognosis of GC, including DNA repair genes, have attracted growing attention in recent years in the field of oncology^10–12^. Impaired genome stability and mutation are a hallmark of cancer that participates in the initiation and progression of malignancies^13,14^. Cells develop multiple kinds of complex DNA repair mechanisms to repair DNA damage, including DNA damage response (DDR), and to maintain genomic integrity. DNA repair acts constantly in human cells to recognize and correct damage to the DNA molecules that encode its genome^15^. Disorders in DDR process is closely correlated with failure to accurately repair damaged DNA in cells, which contributes to the transformation of normal cells into tumor cells with accumulated genetic changes^16^. Recently, researchers have demonstrated the relationship between the aberrant expression of DNA repair genes with the cancer initiation, progression, and prognosis^12,17,18^. However, to our knowledge, there is no currently accurate prognostic signature based on DNA repair genes in GC. Therefore, the present study aimed to construct and validate a prognosis signature based on DNA repair genes via a comprehensive evaluation and further explore its diagnostic value.

## Results

### Differentially expressed DNA repair genes identification and signature construction

**Figure 1:**
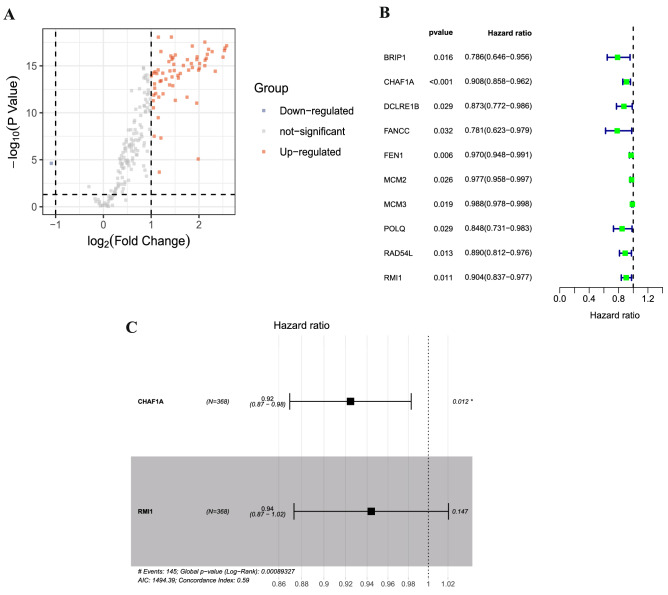
Prognostic DNA repair genes identification and signature construction in the TCGA cohort. (**A**) The volcano plot of the differentially expressed genes between GC and normal samples; (**B**) Univariate Cox regression analysis identifying prognostic variables with HR with 95% CI and *P* values; (**C**) Prognostic signature construction using multivariate Cox regression analysis.

The TCGA cohort contained 368 GC patients with survival information, and patients clinicopathologic characteristics are listed in Table 1. The workflow for present study is illustrated in Supplementary Figure 1. Expression profiles were compared between the GC and normal controls to obtain differentially expressed DNA repair genes. A total of 66 DEGs were identified (Fig. 1A). The univariate Cox regression analysis was performed in these DEGs. We screened a total of 10 genes with prognostic value (Fig. 1B). Then, multivariate Cox regression analysis was carried to construct a risk signature. In total, two DNA repair genes (CHAF1A and RMI1) were incorporated into the model (Fig. 1C) and to evaluate the survival risk of each patient as follows: Risk score = – 0.07858* CHAF1A expression – 0.05766* RMI1 expression. Therefore, we divided the patients into high- and low-risk groups using the median value of risk scores.

**Table 1:** Associations with risk group and clinical characteristics in the TCGA cohort.

Characteristic	TCGA cohort (N = 368)	high-risk group (N = 184)	low-risk group (N = 184)	*P* value
Age (years)	< 60	61	49	0.099
	> = 60	123	132	
	NA	0	3	
Gender	Female	61	72	0.233
	Male	123	112	
Tumor Stage	Stage I	22	27	0.798
	Stage II	54	56	
	Stage III	75	73	
	Stage IV	19	19	
	NA	14	9	
Neoplasm	Yes	41	34	0.537
	No	116	126	
	NA	27	24	
Survival Status	Living	98	125	0.004
	Dead	86	59	
Grade	Grade I	6	4	0.104
	Grade II	66	67	
	Grade III	104	112	
	NA	8	1	
Family history of GC	Yes	6	9	0.723 1
	No	134	133	
	NA	44	42	
Prior cancer	Yes	5	5	
	No	179	179	
Helicobacter pylori infection	Yes	10	8	0.271
	No	64	79	
	NA	110	97

### Prognostic signature evaluation and external validation

**Figure 2:**
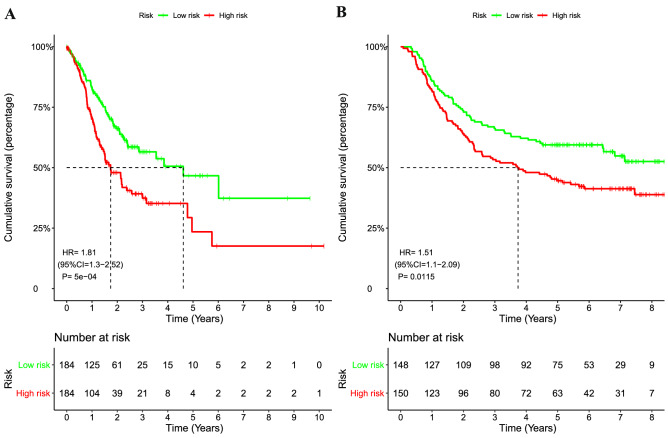
Kaplan–Meier survival analysis of the signature risk score between the high- and low-risk groups. Survival differences in the TCGA cohort (**A**), and the GSE66229 validation cohort (**B**).

**Figure 3:**
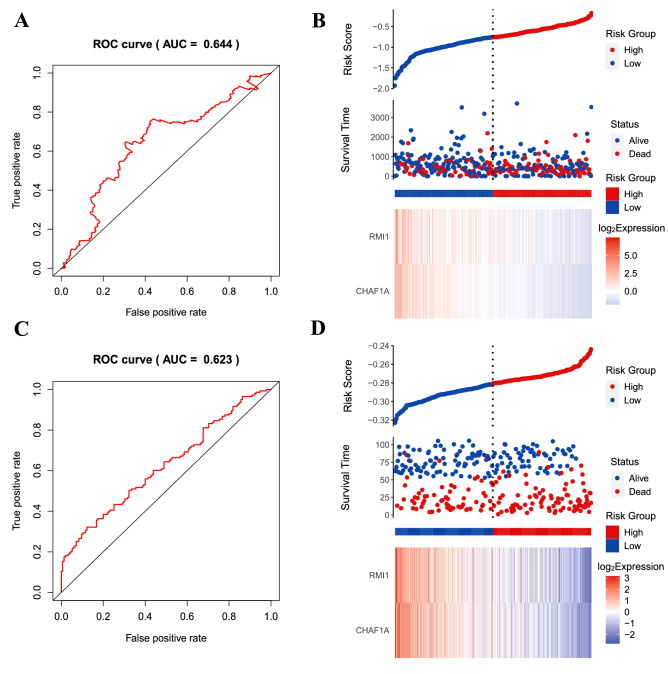
Prognostic value of the two genes signature for prediction of overall survival of patients with GC. (**A**) ROC curve analysis for predicting survival in patients with GC according to the risk score in the TCGA cohort; (**B**) From top to bottom are the risk score, patients’ survival status distribution, and the expression heat map of two genes in the low- and high-risk groups in the TCGA cohort; (**C**) ROC curve analysis for predicting survival in patients with GC according to the risk score in the GSE66229 cohort; (**D**) From top to bottom are the risk score, patients’ survival status distribution, and the expression heat map of two genes in the low- and high-risk groups in the GSE66229 cohort. A heat map was generated using the “pheatmap” package (version 1.0.12; https://cran.r-project.org/web/packages/pheatmap/index.html) of the R software (version 3.6.3).

As revealed in the Fig. 2A, GC patients in high-risk group demonstrated a significantly unfavorable OS than patients in low-risk group in the TCGA cohort (HR = 1.81, 95%CI = 1.3–2.52, *P* < 0.0001), and further validated in the GSE66229 dataset (HR = 1.51, 95%CI = 1.1–2.09, *P* = 0.0115; Fig. 2B). The result of time-dependent ROC curve analysis revealed that the novel signature could accurately predict the OS of patients with GC (Fig. 3A). As demonstrated in Fig. 3B, a heatmap was shown to present the expression profile of the two genes. The patients were sorted according to risk score and classified into high- and low-risk groups. As the risk score of patients with GC increased, the number of patients deaths elevated. A chi-squared test revealed that the mortality rate of the high-risk group was significant high than that in the low-risk groups (46.7 vs 32.1%, *P* = 0.004). Similar results were observed in the validation cohort (Fig. 3C-D). In GSE66229 cohort, the AUC was 0.623 and survival analysis revealed a favorable performance of the signature for stratifying high-risk and low-risk patients. The mortality rate of the high-risk group was 58.7%, which was high than that of the low-risk group of 43.2% (*P* = 0.0078).

### Risk score of the two gene signature as an independent factor for predicting GC Prognosis

A univariate Cox regression analysis was firstly performed among all available clinicopathological variables in the TCGA cohort to determine whether the risk score was an independent prognostic factor for OS. The univariate Cox proportional hazards regression analysis result illustrated that high risk was significantly associated with shorter survival in the TCGA cohort (HR = 2.145, 95%CI = 1.249- 3.685; *P* = 0.0057; Table 2). The risk score remained as an independent prognostic predictor in the multivariate analyses, after adjusting other clinicopathological variables (HR = 2.313, 95%CI = 1.276–4.193; *P* = 0.0057). Furthermore, the independent prognostic factor was confirmed in the GSE66229 cohort (HR = 1.459, 95%CI = 1.048–2.029; *P* = 0.0251). This suggested that the two gene signature has good independence in clinical application.

**Table 2:** Univariate and multivariate analyses identified independent prognostic factors for overall survival of patients with GC in the TCGA cohort and GSE66229 dataset.

	Univariate analysis			Multivariate analysis		
	HR	95%CI	*P* value	HR	95%CI	*P* value
**TCGA cohort**						
riskScore (high vs low)	2.145	1.249–3.685	0.0057	2.313	1.276–4.193	0.0057
Age (continuous)	1.032	1.003–1.062	0.0325	1.05	1.014–1.088	0.0066
Sex (Female/Male)	1.541	0.849–2.793	0.1544	1.142	0.609–2.141	0.6776
Stage (I/II/III/IV)	1.758	1.218–2.539	0.0026	1.453	0.992–2.128	0.0552
Grade (I/II/III/IV)	1.466	0.898–2.392	0.1258	1.515	0.893–2.568	0.1231
Neoplasm status (Yes/No)	5.585	3.132–9.956	< 0.0001	4.493	2.386–8.458	< 0.0001
Family history of GC (Yes/No)	1.056	0.477–2.336	0.8929	0.939	0.403–2.191	0.886
Prior cancer (Yes/No)	1.187	0.288–4.891	0.8121	2.856	0.626–13.029	0.1754
Helicobacter pylori infection (Yes/No)	0.912	0.385–2.16	0.8333	0.571	0.218–1.496	0.2544
**GSE66229 cohort**						
riskScore (high vs low)	1.515	1.098–2.091	0.0115	1.459	1.048–2.029	0.0251
Sex (Female/Male)	0.917	0.656–1.282	0.6114	1.056	0.752–1.482	0.7539
Age (continuous)	1.011	0.996–1.026	0.1566	1.026	1.011–1.043	0.0013
Stage (I/II/III/IV)	2.215	1.826–2.686	< 0.0001	2.314	1.895–2.827	< 0.0001

### Nomogram construction based on the signature

**Figure 4:**
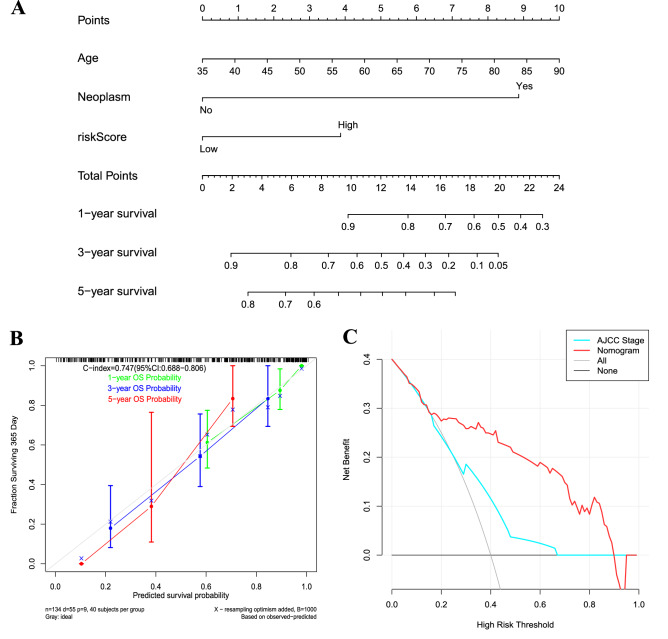
Nomogram construction based on the DNA repair gene signature. (**A**) Nomogram predicting overall survival probability for patients with GC; Assign the points of each variable of the patient by drawing a vertical line from that variable to the points scale, next, sum all the points, and draw a vertical line from the total points’ scale to the 1-, 3-, and 5-year OS to obtain the probability of death. (**B**) Calibration plots for the nomogram; Nomogram-predicted OS is plotted on the x-axis, and actual OS is plotted on the y-axis. A plot along the 45° line would present a perfect calibration model in which the predicted probabilities are identical to the actual outcomes. (**C**) decision curve analyses comparing nomogram and AJCC stage; the net benefit was plotted versus the threshold probability.

A nomogram incorporating the independent factors, age, neoplasm, and risk score, was built to predict 1-, 3-, and 5-year OS (Fig. 4A). The C-index for TNM stage and the nomogram (combined model) was 0.747 (95% CI: 0.688–0.806), and 0.634 (95% CI: 0.564–0.704), respectively. Calibration plots presented an excellent agreement between the prediction based on the nomogram and the actual observations (Fig. 4B). According to decision curve analyses, the nomogram also offered the highest net benefit than the TNM stage examined (Fig. 4C).

### Diagnostic classifier based on genes signature

**Figure 5:**
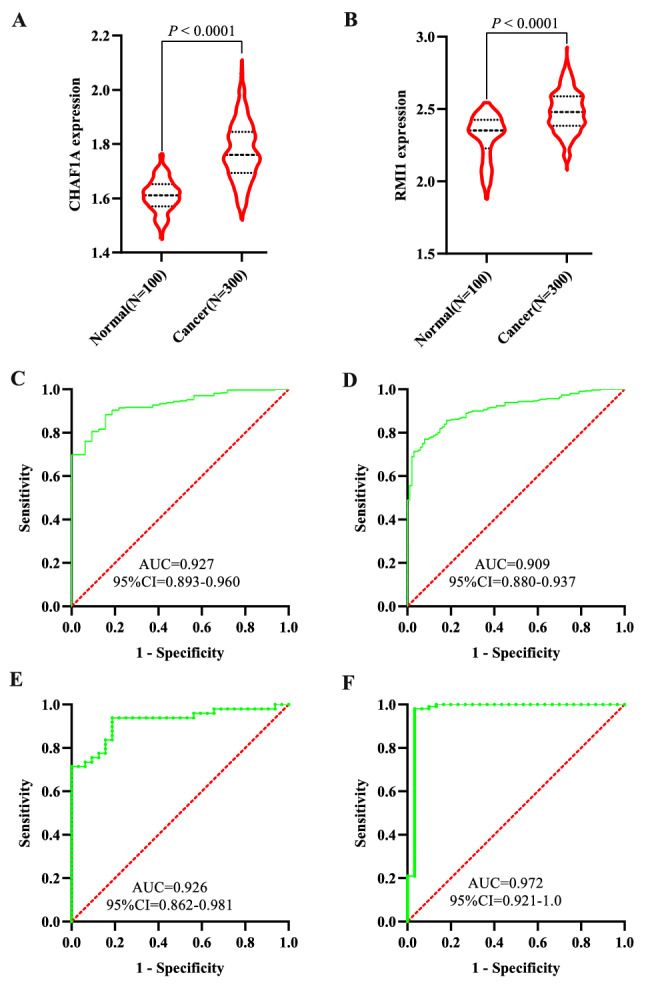
Validation of expression pattern of two identified genes in the validation cohort and the diagnostic performance of signature genes in distinguishing GC from normal samples. The expression changes of CHAF1A (**A**) and RMI1 (**B**) in the GSE66229 cohort; The ROC curves of the two genes-based diagnostic classifier in the TCGA cohort (**C**) and the independent GSE66229 cohort (**D**); ROC curves of the diagnostic classifier for stage I patients with GC in the TCGA cohort (**E**) and the GSE66229 cohort (**F**).

First, the expression patterns of the two genes in the signature were further validated in the GSE66229 cohort at the mRNA level. CHAF1A and RMI1 expression are remarkably higher in tumor tissues of GC when compared with normal samples (all *P* < 0.0001; Fig. 5A,B). Next, CHAF1A and RMI1were selected for multivariate logistic regression analysis to obtain diagnostic score. The ROC curves for combined diagnosis in terms of diagnostic score illustrated high accuracy in distinguishing GC patients from normal controls in the TCGA cohort with an AUC of 0.927 (95%CI = 0.893–0.960; Fig. 5C). Moreover, we evaluated the ability of the diagnostic classifier in differentiating between GC and control tissues, demonstrating that the model also had a high accuracy of prediction (AUC = 0.909, 95%CI = 0.880–0.937) in the GSE66229 cohort; Fig. 5D). As for early diagnosis of GC, the results of stratification analyses for the stage I group in TCGA dataset (AUC = 0.926, 95%CI = 0.862–0.981; Fig. 5E), and the stage I group in the GSE66229 cohort (AUC = 0.972, 95%CI = 0.921–1.0; Fig. 5F) demonstrated the robust diagnostic performance of the diagnostic classifier based on genes signature. These data further confirmed that the diagnostic classifier was a novel predictive tool with high accuracy and potential clinical value.

### Association between the identified genes and tumor-infiltrating immune cells

**Figure 6:**
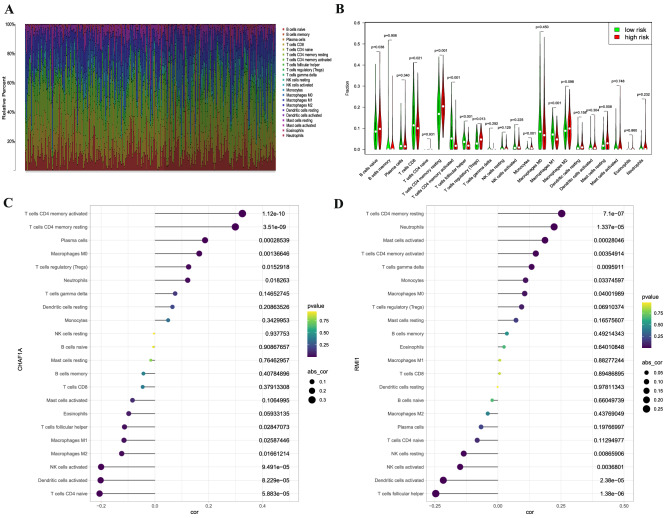
Distribution and visualization of immune cell infiltration in patients with GC and the correlation between two DNA repair genes. Summary of estimated compositions of 22 immune cell subtypes from the CIBERSORT algorithm in GC patients (**A**); Comparison of 22 immune cell subtypes between low- and high-risk samples (**B**). The correlation between CHAF1A (**C**) and RMI1 (**D**) and infiltrating immune cells in patients with GC.

CIBERSORT algorithm was performed to estimate the relative abundance of 22 kinds of immune cells for each sample and compared between the high and low-risk group. The abundance ratios of 22 types of immune cells in the GC samples was calculated (Fig. 6A). The proportions of B cells naïve (*P* = 0.038), resting CD4 memory T cells (*P* < 0.001), T cells regulatory (*P* = 0.013), monocytes (*P* < 0.001), and resting mast cells (*P* = 0.008) in high-risk group were significantly higher than in low-risk group. However, the proportion of CD8 T cells (*P* = 0.021), activated CD4 memory T cells (*P* < 0.001), follicular helper T cells (*P* < 0.001), and M1 macrophages (*P* < 0.001) in high-risk group were significantly lower than in low-risk group (Fig. 6B).

The correlation between the expression of CHAF1A and RMI1 and immune cells infiltrating in GC was evaluated by Spearman's correlation. As revealed in Fig. 6C, CHAF1A was positively correlated with activated CD4 memory T cells (r = 0.325, *P* = 1.12E−10), resting CD4 memory T cells (r = 0.299, *P* = 3.51E−09), plasma cells (r = 0.186, *P* = 0.00029), M0 macrophages (r = 0.165, *P* = 0.0014), T cells regulatory (r = 0.125, *P* = 0.0153), and neutrophils (r = 0.122, *P* = 0.0182), and negatively correlated with naive CD4 T cells (r =  − 0.206, *P* = 5.88E−05), activated dendritic cells (r =  − 0.202, *P* = 8.23E−05), activated NK cells (r =  − 0.200, *P* = 9.49E−05), M2 macrophages (r =  − 0.124, *P* = 0.0166), M1 macrophages (r =  − 0.115, *P* = 0.0259), and follicular helper T cells (r =  − 0.113, *P* = 0.0285). RMI1 was positively correlated with resting CD4 memory T cells (r = 0.253, *P* = 7.07E−07), neutrophils (r = 0.223, *P* = 1.34E−05), activated mast cells (r = 0.187, *P* = 0.00028), activated CD4 memory T cells (r = 0.150, *P* = 0.0035), gamma delta T cell (r = 0.134, *P* = 0.0096), monocytes (r = 0.110, *P* = 0.0337), M0 macrophages (r = 0.106, *P* = 0.04), and negatively correlated with follicular helper T cells (r =  − 0.246, *P* = 1.38E−06), activated dendritic cells (r =  − 0.216, *P* = 2.38E−05), activated NK cells (r =  − 0.150, *P* = 0.0037), and resting NK cells (r =  − 0.135, *P* = 0.0087; Fig. 6D).

### Gene set enrichment analyses

**Figure 7:**
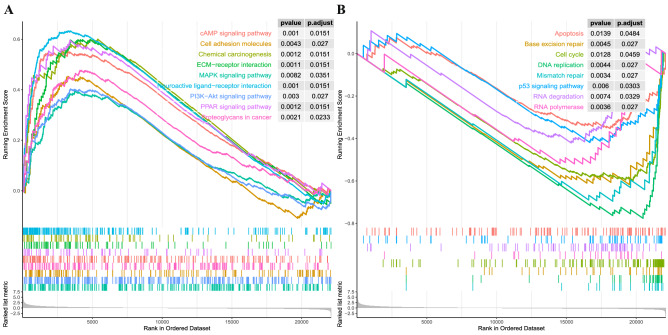
GSEA illustrated the significantly altered biological processes in high-risk group and low-risk group in the TCGA cohort.

As demonstrated in the Fig. 7A, cMAP signaling pathway, cell adhesion molecules, ECM-receptor interaction, proteoglycans in cancer, MAPK, PRAR, and PI3K-Akt signaling pathways were significantly enriched in the high-risk group. Cell cycle, DNA replication, mismatch repair, apoptosis, RNA degradation, base excision repair, and p53 signaling pathways were significantly enriched in the low-risk group (Fig. 7B).

## Discussion

GC remains a major commonly diagnosed malignancy worldwide especially in Asian countries^19^. It was known that survival prediction affects the choices of multiple treatment options, thus more efforts are required to achieve a favorable prognosis for GC, which have been considered as a major challenge for the clinical use. Accurate prediction of prognosis and early diagnosis are important for GC to achieve accurate individualized treatment. Currently, the assessment of cancer prognosis relies on the well-known useful and common TNM staging^20^. However, TNM staging is not able to completely predict the prognosis of patients and there is an urgent need for other biomarkers to help and supplement the TNM staging. In recent years, high-throughput sequencing technology and bioinformatics analysis has been widely used to identify candidate genes related to various diseases that might act as diagnostic and prognostic biological markers^8,9,11,21^. Increasing evidences has demonstrated the roles of DNA damage and repair genes in cancers, including GC^22,23^. However, up to now, there was no prognostic signature based on DNA repair genes and its prospective diagnostic value has been reported in GC. Thus, we developed a two-DNA repair genes based signature predicting the OS of patients with GC.

In this study, we used a high-throughput method to identify DNA repair genes associated with GC prognosis and conducted a comprehensive analysis to develop a prognostic signature for GC survival prediction and early diagnosis. Ten prognosis genes were remained after univariable Cox regression, which were then subjected to multivariate Cox regression analysis. Finally, a two-gene signature was generated and validated its efficiency in a validation cohort, which both could successfully assign patients into low-risk and high-risk groups with distinct OS, where patients in the high-risk group demonstrated a significantly poor prognosis than the low-risk group, which providing a basis for further precision treatment. The signature also demonstrated to be an independent prognosis factor for GC survival in two cohorts. A nomogram combining age, neoplasm, and risk score was established, which proved to be a better predictor than nomogram constructed with TNM stage. Thus, the signature composed of the two DNA repair genes could be an effective predictor for GC prognosis and contribute to the prognosis prediction. Furthermore, the logistic regression-based diagnostic classifier incorporating the two genes demonstrated perfect discriminatory ability in distinguishing GC from normal tissues with an AUC of 0.927 (95%CI = 0.893–0.960) in the TCGA cohort and an AUC of 0.909 (95%CI = 0.880–0.937) in the validation cohort. Moreover, the diagnostic classifier also showed a perfect diagnosis performance for GC patients in early stage with an AUC of 0.926 (95%CI = 0.862–0.981) in the TCGA cohort and validated in the GSE66229 dataset with an AUC of 0.972 (95%CI = 0.921–1.0). These results revealed that the signature could provide an accurate prognosis as well as early diagnosis for patients with GC. What's more, GSEA exhibited multiple gene sets from numerous molecular signatures respectively enriched in the high- or low-risk group, which might account for the possible mechanism of the two-gene based signature.

The two signature genes identified have been previously validated in multiple types of cancers. Chromatin assembly factor-1 (CAF-1), which consisting of p48, p60 and p150 (CHAF1A) subunits, plays a vital role in various biological processes, such as DNA replication during the nucleosome formation and the chromatin restoration after DNA repair^24–26^. CHAF1A (CAF p150), the main functional subunit of CAF-1, promotes rapid assembly of nucleosomes on newly replicated DNA, and involved in DNA replication, gene expression regulation and DNA mismatch repair^25,27,28^. CHAF1A plays a vital role in contributing to the occurrence and development of malignancies. Increasing reports have found that CHAF1A was closely associated with cell cycle regulation and showed a pivotal relationship with the formation and prognosis of various cancers, which can served as a biomarker to distinguish quiescent from proliferating cells^29^. Recently, CHAF1A has been revealed to be upregulated and associated with cell differentiation, proliferation, and apoptosis resistance in multiple cancers, including GC^30,31^. Our results coincided with previous study that CHAF1A was reported overexpressed in GC cell lines and tissue samples and its high expression was predictive of poor survival. Functional in vitro studies manifested that its expression contributed to GC cell proliferation by strengthening transcriptional activation of c-MYC and CCND1 genes in concert with TCF4^30^. DNA replication is indispensable to maintain DNA integrity and suppress cancer predisposition. Preservation of chromosome integrity is essential for the viability and fitness of all living cells and organisms and DNA instability usually results in tumorigenesis^32^. RecQ-mediated genome instability protein 1 (RMI1), together with topoisomerase IIIa (Topo IIIa), Bloom’s syndrome helicase (BLM), forms a conserved BTR complex and its absence causes genome instability^33^. Previous studies demonstrated that RMI1 participates in maintaining chromosome stability through responses to DNA double-strand breaks, DNA resection reactions, and replication stress^34–36^. Knockdown of RMI1 damages DNA repair under DNA replication stress, which could account for the molecular basis for its function in maintaining genome integrity^35^. Tumor-infiltrating immune cells have a high prognostic value as to tumor progression and patient’s survival in many solid organ malignancies, including GC^37^. These results were concordant with the findings in our study. We found that the two genes were correlated with multiple tumor-infiltrating immune cells. CHAF1A and RMI1 were shared correlated with activated memory CD4T cells, resting memory CD4T cells, activated dendritic cells, activated NK cells, M0 macrophages, neutrophils, and follicular helper T cells.

To our knowledge, this is the first study to establish a prognostic signature based on DNA repair genes in GC. Nevertheless, the study had some limitations. As a retrospective study, the study has shortcomings associated with retrospective data collected from the TCGA and GEO databases. Therefore, large-scale multicenter prospective cohorts are needed for external validation. In addition, future in vitro and in vivo experiments should be performed to further confirm the findings.

## Conclusion

In this study, a novel two-DNA repair gene signature (CHAF1A and RMI1) was successfully constructed to predict the survival of patients with GC. Moreover, the novel signature is an independent risk factor associated with GC. The signature could not only act as a novel biomarker for the risk stratification of GC patients, but also serve as a diagnostic classifier for the early diagnosis for GC. The signature is closely correlated with immune cell infiltration, which may be a useful prediction tool to identify patients who will benefit from immunotherapy.

## Methods

### Data source and DNA repair genes acquisition

The level 3 mRNA expression data as well as related clinical follow-up information of GC were downloaded from TCGA-GDC database (https://portal.gdc.cancer.gov/), containing 375 GC and 32 adjacent gastric tissues. Transcript expression was calculated as FPKM. The probe IDs were changed into the corresponding gene symbols based on their annotation files. When several probes matched to an identical gene symbol, we averaged them for further analysis. We obtained 727 DNA repair genes from the KEGG portal (https://www.kegg.jp/) and the previous literature (Supplementary Table S1)^21^. Moreover, an independent dataset, GSE66229 (N = 400) and corresponding clinical information used for validation, was downloaded from the GEO database. The GSE66229 dataset contains 300 GC samples and 100 adjacent gastric tissues. We used the GSE66229 dataset as the validation cohort to validate the prognostic signature. The overlapping 210 genes among the two cohorts were used for subsequent analysis.

### Screening of differentially expressed DNA repair genes

Limma package in R computing environment was applied to identify the differentially expressed DNA repair genes (DEGs) between GC and normal gastric tissues^38^. Next, we performed gene differential analysis with the threshold of absolute value of the log2 fold change (logFC) > 1 and false discovery rate (FDR) < 0.05 in the TCGA cohort. The integrated DEGs lists were used for subsequent analysis.

### Prognostic DNA repair genes identification and signature establishment

Univariate and multivariate Cox regression analyses were performed to analyze the relationship of DEGs with OS in GC in the TCGA cohort. The univariate Cox regression analysis of the DEGs was screened using R “survival” package. DEGs with *P* value < 0.05 were regarded as candidate genes. Multivariate analysis was used to identify the best model according to the smallest Akaike Information Criterion (AIC) value, which is a measure of the goodness of fit^39^. Ultimately, a prognostic signature was constructed by the multiplication of gene expression and regression coefficient (β) according to the following equation: *Risk score* = *βgene*_*1*_ * gene_*1*_* expression* + *βgene*_*2*_ * gene_*2*_* expression* + *·····* + *βgene*_*n*_ * gene_*n*_* expression.* Based on the formula, we calculated the signature risk score of all patients. GC patients were classified into high-risk and low-risk groups for further study according to the median value of the risk score. Kaplan–Meier analysis was performed to compare the statistical differences in survival rate between the high-risk and low-risk groups. Furthermore, we performed time-dependent receiver operating characteristic (ROC) curve analysis with an R package“survivalROC” to evaluate the predictive accuracy of the prognostic signature. The area under the curve (AUC) was computed to measure the predictive ability of the gene signature.

### DNA repair genes signature for prediction independent of other clinical characteristics

The DNA repair genes signature together with other available clinical characteristics including age, sex, grade, TNM stage, neoplasm status, family history of GC, prior cancer, and Helicobacter pylori infection were subjected to the univariate Cox regression analyses. Then, variables associated with OS were putted into the multivariate Cox regression model to determine whether the signature was an independent prognostic predicator of OS in GC.

### Validation of gene expression pattern and prognostic signature

GSE66229 dataset was used for the validation of identified DEGs. The risk score of each patient was computed based on the same risk formula mentioned above and patients were grouped into the high- or low-risk subgroups according to the median risk score. The same analyses were conducted to validate the reliability and validity of the signature, including Kaplan–Meier analysis, ROC curve analysis, and multivariate Cox proportional hazards analysis.

### Constructing and validating a predictive nomogram

A nomogram was formulated on the basis of the prognostic factors determined by the multivariate Cox proportional hazards regression analysis to generate an individual prediction of OS using the “rms” package in R software. Validation of the nomogram was explored by discrimination and calibration. Harrell’s concordance index (C-index) was calculated to assess the predictive accuracy of the model by a bootstrap method and to compare with the AJCC TNM staging system. Furthermore, we plotted decision curve analysis (DCA) curves to explore the benefits of nomogram-assisted decisions in a clinical context and compared with the AJCC staging system. The optimal model is the one with the highest net benefit as calculated.

### Area under receiver operating characteristic analysis to explore the diagnostic performance of the signature for GC

To evaluate the diagnostic performance of the signature in distinguishing GC patients from normal controls, ROC analysis of each identified gene was performed between 375 patients with GC and 32 normal controls in the TCGA cohort and further validated in the GSE66229 dataset, which included 300 HCC and 100 adjacent normal samples. We formulated a diagnostic model with identified genes by using the logistic regression analysis to distinguish GC from normal tissue. In this model, the diagnostic scores were evaluated as continuous variables.

### Estimation of the immune landscape and correlation analysis

To distinguish the relative proportions of infiltrating immune cells from the gene expression profiles in GC, CIBERSORT (https://cibersortx.stanford.edu/) was used to deduce the 22 immune cell scores in the TCGA cohort by comparing the proportion of samples with the expression of Leukocyte signature matrix (LM22) signature genes using the R package “corrplot” with 1000 permutations^40^. Cases with a CIBERSORT output of *P* < 0.05 were selected for the next analysis. Violin plots were drawn using the “vioplot” package in R to visualize the differences in immune cell infiltration between the high-risk and low-risk groups. The association of the identified gene biomarkers with the levels of infiltrating immune cells was explored using Spearman’s rank correlation analysis in R software. The resulting associations were visualized using the chart technique with “ggplot2” package.

### Gene set enrichment analysis

Gene set enrichment analysis (GSEA) was carried out to investigate whether a priori defined set of genes presented significant differential expression between the high- and low-risk risk groups in the enrichment of MSigDB Collection^41^. The risk score was used as a phenotype label. The nominal *P* value and normalized enrichment score (NES) were evaluated to sort the pathways enriched in each phenotype. Gene set permutations for each analysis were executed 1000 times. An absolute value of the standardized NES > 1 and a nominal *P* value of less than 0.05 were regarded as the threshold of statistical significance. Gene sets at *P* < 0.05 was considered to be significantly enriched and to identify biological processes.

### Statistical analysis

The expression patterns of identified genes between GC and normal samples were compared using student’s t test. A heat map was generated using the “pheatmap” package (version 1.0.12) of the R software (version 3.6.3). The diagnostic and prognostic prediction models were analyzed by ROC curve and time-dependent ROC curve, respectively, and quantified by the AUC. A *P* value < 0.05 was considered to be significant. All statistical analyses were performed using R (version 3.6.3; https://www.r-project.org/).

### Informed consent

Written informed consent was waived since all data are from public databases.

## Supplementary Information

Supplementary Figure 1 The flowchart of the study design to establish and verify of the prognostic signature.

Supplementary Information 2.

## Data Availability

The data sets involved in our study are publicly available in GEO database (https://www.ncbi.nlm.nih.gov/geo/) and the TCGA database (https://portal.gdc.cancer.gov/).
