# Supplementary figures and images for: Identification and external validation of a prognostic signature associated with DNA repair genes in gastric cancer
Source: Sci Rep. 2021 Mar 30;11:7141. doi: 10.1038/s41598-021-86504-8 (PMC8010105; doi:10.1038/s41598-021-86504-8)

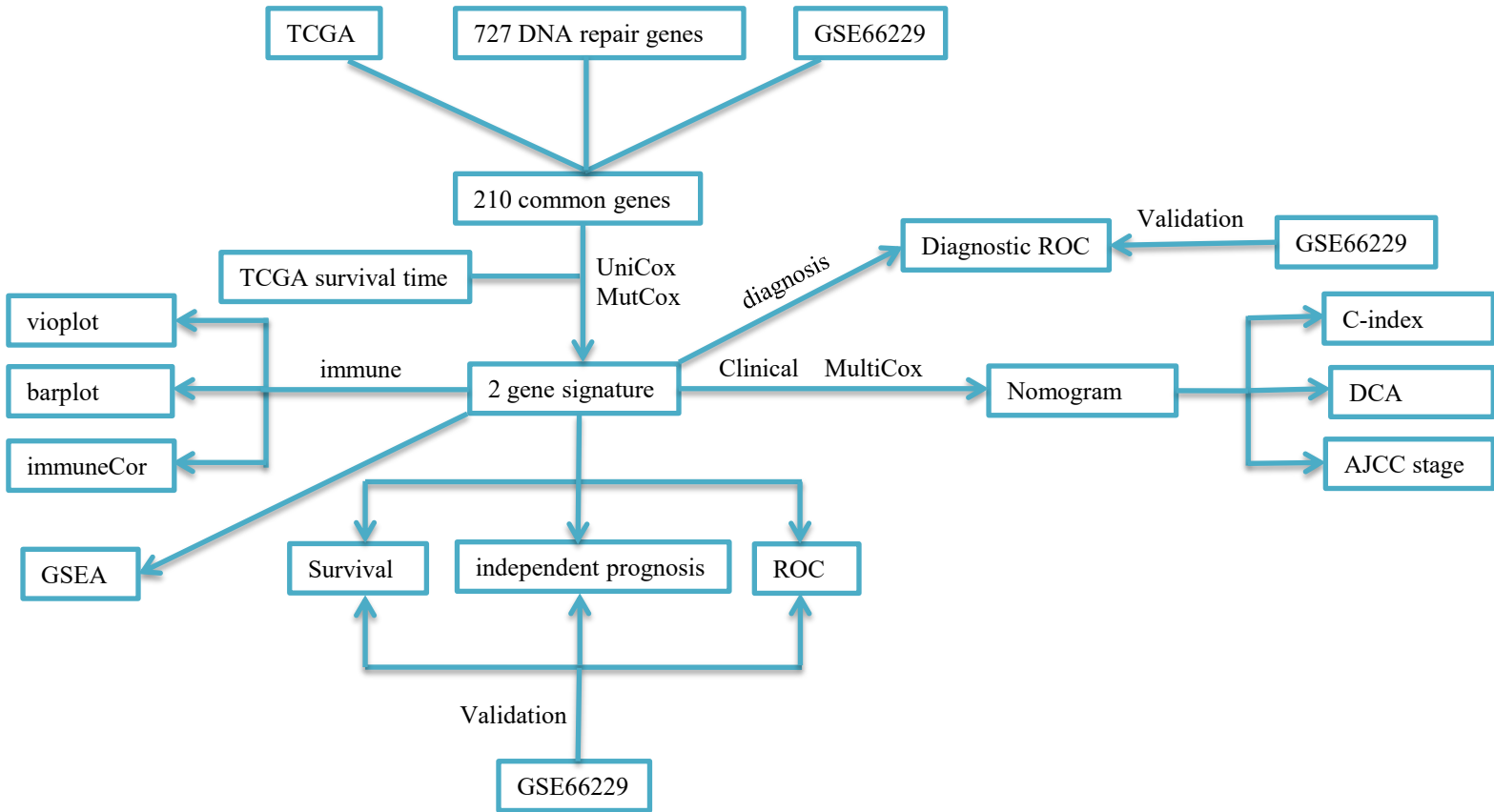

Supplement: Supplementary file 1 — Supplementary Figure 1 The flowchart of the study design to establish and verify of the prognostic signature. [file 41598_2021_86504_MOESM1_ESM.pdf]
