# Supplementary material for: Identification and external validation of a prognostic signature associated with DNA repair genes in gastric cancer
Source: Sci Rep. 2021 Mar 30;11:7141. doi: 10.1038/s41598-021-86504-8 (PMC8010105; doi:10.1038/s41598-021-86504-8)
Supplement: Supplementary file 2 — Supplementary Information 2. [file 41598_2021_86504_MOESM2_ESM.docx]

***Identification and external validation of a prognostic signature associated with DNA repair genes in gastric cancer***

Shimin Chen, Wenbo Liu, Yu Huang*

Department of Gastroenterology, Traditional Chinese Medical Hospital of Taihe Country, Taihe, 236600, China

Corresponding authors:

Yu Huang; Department of Gastroenterology, Traditional Chinese Medical Hospital of Taihe Country, Taihe, 236600, China; **E-mail:** 554766788@qq.com

***Supplementary Table S1*** 727 DNA repair genes from the KEGG portal (https://www.kegg.jp/) and the previous literature

| A1 | ARTD1 | BRIP1 | CENPS | CUL4B | EME1 | FAAP95 | FANCL | HNPCC | GTF2H2C_2 | MCG40308 |
| --- | --- | --- | --- | --- | --- | --- | --- | --- | --- | --- |
| AAG | ARTD2 | BROVCA1 | CENP-S | CUL-4B | EME2 | FAB | FANCM | HNPCC1 | GTF2H2D | MCM5 |
| ADPG | ARTD3 | BROVCA2 | CENPX | CycH | ENDOV | FAC | FANCN | HNPCC2 | GTF2H3 | MCM6 |
| ADPRT | ARTD4 | BROVCA3 | CENP-X | D3S3194 | ERCC1 | FACA | FANCO | HNPCC4 | GTF2H4 | MCM7 |
| ADPRT_1 | A-SCID | BROVCA4 | CETN2 | D9 | ERCC11 | FACB | Fanconi | HNPCC5 | GTF2H5 | MDG |
| ADPRT1 | ATLD | BS | CHAF1A | DCLRE1A | ERCC2 | FACC | FANCP | HNPCC7 | GTMBP | MDPL |
| ADPRT2 | ATLD2 | BTBD12 | CHARAC17 | DCLRE1B | ERCC3 | FACD | FANCQ | hNTH1 | H1RNA | MEC1 |
| ADPRT3 | ATM | BTF2 | CHEK1 | DCLRE1C | ERCC4 | FACE | FANCR | HOGG1 | H2AFX | MED1 |
| ADPRTL1 | ATR | BTF2P44 | CHEK2 | DCLREC1C | ERCC5 | FAD | FANCS | HR23A | HAP1 | MF1 |
| ADPRTL2 | ATRIP | C16orf75 | CHRAC17 | DDB1 | ERCC5-201 | FAD1 | FANCT | HR23B | HCAK | MGMT |
| ADPRTL3 | ATV | C17orf70 | CKN1 | DDB2 | ERCC5-202 | FAD2 | FAP3 | HR54 | HCC5 | MHCBFB |
| AGS2 | AT-V1 | C19orf40 | CKN2 | DDBA | ERCC6 | FA-D2 | FCC1 | hRad50 | hCdc21 | MHF1 |
| AGS3 | AT-V2 | C6orf175 | CLK2 | DDBB | ERCC8 | FAE | FCC2 | HRAD51 | hDNA2 | MHF2 |
| AGS4 | AYP1 | C9orf76 | CMM6 | DGU | ERCM2 | FAF | FCTCS | hRAD54 | HELQ | Mid1 |
| ALKBH2 | bA120J8.2 | CAK | COCA1 | DINB1 | EXO1 | FAG | FDG | HRY | HES1 | Mis5 |
| ALKBH3 | BA554C12.1 | CAK1 | COCA2 | DINP | FA | FAH | FEN1 | HSAP | HES-1 | MITOTIN |
| anpg | BACH1 | CALT | COFS | DLEU8 | FA1 | FA-H | FEN-1 | HSPC150 | HEX1 | XPD |
| APE | BETAN | CAP35 | COFS1 | DMC1 | FA2 | FAN1 | FGP2 | HsRad51 | hExoI | XPE |
| APE1 | bHLHb39 | CCNH | COFS2 | DNA2 | FA3 | FANCA | FILS | HSSB | hFAN1 | XPE-BF |
| APE2 | BIVM-ERCC5 | CCNL1 | COFS3 | DNA2L | FA4 | FANCB | FPG1 | HsT16930 | hFPG1 | XPF |
| APEN | BLAP18 | cdc19 | COFS4 | DNAPK | FAA | FANCC | FPG2 | HSU24186 | hFPG2 | XPG |
| APEX | BLAP75 | CDC2 | COR1 | DNA-PKcs | FAAP10 | FANCD | FRP1 | hTDG | HHL | XPGC |
| APEX1 | BLM | CDC21 | CRA36.1 | DNPK1 | FAAP100 | FANCD1 | G22P1 | HUS1 | HHR23A | XPV |
| APEX2 | BM28 | CDC46 | CRCS10 | DNTT | FAAP16 | FANCD2 | GEN1 | HYRC | HHR23B | XP-V |
| APEXL2 | BRCA1 | CDC47 | CRCS12 | DPE2 | FAAP20 | FANCE | GIYD1 | HYRC1 | hHR54 | RMI2 |
| APITD1 | BRCA2 | CDC54 | CSA | DSS1 | FAAP24 | FANCF | GIYD2 | IMD26 | HIGM4 | RNASEH1 |
| APLF | BRCAI | CDCL1 | CSB | DUP | FAAP250 | FANCG | GLM3 | IRIS | HIGM5 | RFC140 |
| APNG | BRCC1 | CDK7 | CTC75 | DUT | FAAP43 | FANCH | gs125 | IRT1 | HLTF | MUTM |
| APTX | BRCC2 | CDKN7 | CTCBF | ECD | FAAP75 | FANCI | GTBP | JUNB | HMG1 | MUTYH |
| APX | BRCC5 | CEN2 | CUL4A | EM9 | FAAP90 | FANCJ | GTF2H | KARP1 | HMG-1 | PMSL2 |
| ARMD5 | NEIL1 | NER-related | NHEJ1 | hNEI3 | HMMH | KMIN | GTF2H1 | KARP-1 | HMG3 | PNAS146 |
| NEIL3 | NEIL2 | NFIV | NKCD | HNGS1 | HMUDG | KU70 | GTF2H2 | KIAA1018 | HMGB1 | POLM |
| TLAA | TTD3 | UVSS1 | UV20 | TELO2 | TP53 | UBE2A | UBP | UVSSA | XP3 | POLN |
| TOP3 | TTDA | UVSS2 | TTDN1 | TFB1 | TP53BP1 | UBE2B | UDG | VAULT3 | XPA | R51H3 |
| TOP3A | TTD-A | TOP3B | UAF1 | TFB2 | TRAD | UBE2N | UNG | VPARP | XPAC | RAD1 |
| XFEPS | TOPBP1 | YHHQ1 | STRA13 | TFB3 | TREX1 | UBE2T | UNG1 | VWA5C | XPB | RCC |
| XLF | ZGRF3 | ZGRF2 | RPA4 | TFB4 | TREX2 | UBE2V2 | UNG15 | WDR48 | XPC | RDH54 |
| XP1 | ZGRF7 | SLX4 | SETMAR | TFB5 | TTD | UV-DDB1 | UNG2 | WRN | XPCC | MCM3 |
| XRCC1 | RNH1 | XRCC2 | SSMED | TFIIH | TTD1 | UV-DDB2 | UNG3 | XAB2 | XPCE | MCM4 |
| XRCC11 | RNHIA | SLX3 | STK1 | TGF2H5 | TTD2 | UVDR | USP1 | XAP1 | TOP3B1 | p125 |
| SLX2A | SSBP1 | RPA3 | SCP3 | RECQL | RECQL3 | SCKL | SBP-1 | RRM2B | RPA70 | p160 |
| SLX2B | RPA32 | SEM1 | RECQL4 | RECQL2 | RECQL5 | SCKL1 | SCIDA | RS-SCID | RPRGL4 | P66 |
| REF1 | REPA1 | REPA2 | REPA3 | REV1 | REV1L | REV3 | REV3L | RF-A | RFC | P68 |
| MLH4 | pADPRT-1 | POG | KIAA1596 | RAD23B | MYH | PNCA2 | POLQ | RAD10 | RDM1 | RFC1 |
| MMS19 | pADPRT-2 | PO-GA | KIAA1794 | RAD25 | NBN | PNCA3 | POLZ | RAD17 | REC2 | MDC1 |
| MMS4L | PADPRT-3 | Pol_Mu | GTF2H2C | RAD26 | NBS | PNCA4 | PPOL | RAD18 | RECA | MAT1 |
| MNAT1 | PALB2 | POL4P | hMLH1 | RAD30 | NBS1 | PNKP | PPP1R104 | RAD2 | RECC1 | MBD4 |
| MO15 | PARP | POLA | P80 | RAD30A | NBSLD | NKGCD | PPP1R128 | RAD23A | RECQ2 | MUS81 |
| MPG | PARP1 | POLA1 | P85MCM | RAD30B | SFM2 | SMUG1 | SYCP3 | RNASEH2C | RNHL | PMS2L3 |
| MRE11 | PARP-1 | POLA2 | P95 | RAD3OB | SHFD1 | SNM1C | T-BTF2P44 | RNASEHI | ROC1 | POLL |
| MRE11A | PARP2 | POLB | ML8 | RAD4 | Shfdg1 | SOSS-B1 | TDG | RNF168 | RP-A | R51H2 |
| MRE11B | PARP-2 | POLD | MLH1 | RAD50 | SHFM1 | SPG60 | TDP1 | RNF4 | RP-A_p14 | RBX1 |
| MRMV2 | PARP3 | POLD1 | MLH3 | RAD502 | SHPRH | SPGF4 | TDP2 | RNF53 | RP-A_p32 | MCM2 |
| MRP1 | PARP4 | POLD2 | NEH2 | RAD51 | SHSF1 | SPO11 | TDT | RNF66 | RP-A_p34 | p12 |
| MRXHF2 | PARP-4 | POLD3 | NEI1 | RAD51A | SLX1A | SPRTN | Tdt-N | RNF75 | RPA1 | P62 |
| MRXS15 | PARPL | POLD4 | NEI2 | RAD51B | SLX1B | SSBP | TEL2 | RNF8 | RPA2 | RNASEH2B |
| MRXSC | PCNA | POLDS | NEI3 | RAD51C | RLFB | RFC5 | RFC4 | RFC37 | RFC3 | MAD2L2 |
| MSH2 | PEOB2 | POLE | NSX | RAD51D | RMI1 | RIF1 | RFC40 | RFC38 | RFC36 |  |
| MSH3 | PER1 | POLE1 | NTH1 | RAD51L1 | RFC2 | NUDT1 | p34 | XRCC3 | KU80 |  |
| MSH4 | PH5P | POLE2 | NTHL1 | RAD51L2 | p17 | OBFC2B | p350 | XRCC4 | Ku86 |  |
| MSH5 | PHF9 | POLE3 | PPP1R53 | RAD51L3 | p180 | OCTS3 | p37 | XRCC5 | KUB2 |  |
| MSH6 | PIG11 | POLE4 | PRIM1 | RAD52 | p193 | OF | p39MO15 | XRCC6 | LCFS2 |  |
| MST075 | PIG16 | POLG | PRIM2 | RAD54A | P1-CDC21 | OGG1 | p44 | XRCC7 | LIG1 |  |
| MTMR15 | PIG50 | POLH | PRIM2A | RAD54B | P1-CDC46 | OGH1 | p49 | XRCC9 | LIG2 |  |
| mtSSB | PMS1 | POLI | PRKDC | RAD54L | P1CDC47 | P1.1-MCM3 | P52 | XTH2 | LIG3 |  |
| Mt-SSB | PMS2 | POLK | PRPF19 | RAD9A | P1-MCM3 | P1.h | P58 | YBL1 | LIG4 |  |
| MUS312 | PMS2CL | POLKAPPA | PSCP | RBBP8 | P34 | P105MCM | p58 | RNASEH2A | LIG4S |  |
